# Electrochemical Determination of Dipyrone Using a Cold-Plasma-Treated Graphite Sheet Electrode

**DOI:** 10.1021/acsomega.4c10957

**Published:** 2025-02-05

**Authors:** Jian F. S. Pereira, Patricia Gabrielle C. A. Macilon, Jorge L. A. de Queiroz, Rodrigo A. A. Munoz, Rogério V. Gelamo, Carlos A. Martínez-Huitle, José H. O. Nascimento, Elisama V. Santos

**Affiliations:** † Department of Chemistry, Federal University of Rio Grande do Norte, 59072-970 Natal, RN, Brazil; ‡ Instituto de Química, Universidade Federal de Uberlandia, 38400-902 Uberlândia, MG, Brazil; § Federal University of Mineiro Triangle, 38025-180 38064-200 Uberaba, MG, Brazil; ⊥ Renewable Energies and Environmental Sustainability Research Group, School of Science and Technology, Federal University of Rio Grande do Norte, 59078-970 Natal, RN, Brazil; # Federal Institute of Education of Rio Grande do Norte, Science and Technology, 59078-970 Natal, RN, Brazil

## Abstract

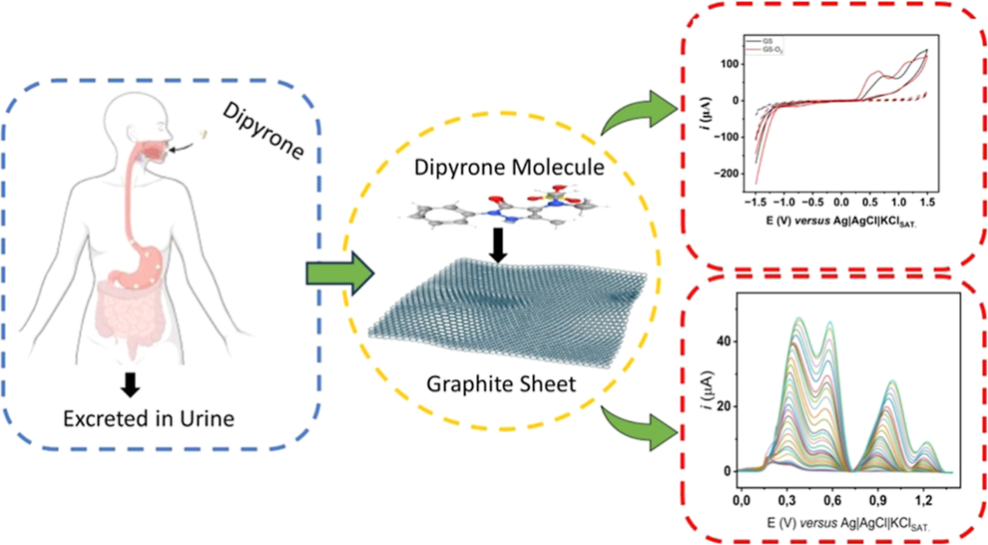

The development of fast, reliable, and cost-effective techniques for pharmaceutical compound analysis is an issue of paramount importance to the pharmaceutical industry, environmental sciences, and many other applications. In this work, a low-cost graphite sheet electrode (GSE) was used as a disposable working electrode. To this purpose, the GSE surface was subjected to a cold plasma discharge using a mixture of argon and O_2_. The sensor was applied to dipyrone (DIP) quantification. Initially, the influence of pH on the electrochemical response of DIP on the pyrolytic graphite sheet (PGS) electrodes was evaluated using a 0.12 mol L^–1^ Britton–Robinson buffer solution at pH values ranging from 2.0 to 12.0. The solution adjusted to pH 4.0 was selected as the supporting electrolyte for the experiments since a larger current intensity was obtained at this medium. The mass transport of DIP toward the PGS surface was investigated by cyclic voltammetry, evidencing a diffusion-controlled process. DIP was initially quantified by square wave voltammetry (SWV) with a linear range of about 2.5–200 μmol L^–1^ and a calculated limit of detection of about 0.31 μmol L^–1^. Finally, SWV was used to enable DIP detection in synthetic urine solutions, demonstrating its applicability as a sensor tool in real analysis.

## 1 Introduction

Dipyrone (DIP, 1-phenyl-2,3-dimethyl-5-pyrazolone-4-methylamino-sodium sulfonate) is pharmacologically classified as a nonsteroidal anti-inflammatory drug with analgesic and antipyretic properties. It is a water-soluble crystalline powder and is also known as metamizole, Novalgin, Analgin, and various other commercial names.^1,2^ It is commonly used to treat colic pain, cancer, migraine pain, and postoperative pain in several countries, such as Russia, Spain, Brazil, and others from South America. However, it has been banned in other countries (for instance, in the USA and UK) due to its association with potentially life-threatening blood dyscrasias, such as agranulocytosis.^3^

Since its abuse can lead to agranulocytosis (incidence of 1 in every 1 million cases), which can later lead to death (in 9% of the cases),^4^ it is important to develop methods to monitor its consumption and presence in human body fluids, such as blood, saliva, and urine. Although DIP hydrolyzes fast when administered, its total absorption ranges from 54% to 89% depending on the administration method, which means that up to 46% of the administered dose is excreted in urine.^5^ Several works have determined DIP by using liquid chromatography,^6,7^ capillary electrophoresis,^8^ and electrochemical methods, such as amperometric^9,10^ and voltammetric^11,12^ methods. Among those methods, electrochemical analysis presents advantages, such as rapid analysis, relatively low cost, and portability.

Several electrodes are reported in the literature to detect DIP, such as a glassy carbon electrode,^13^ boron-doped diamond,^14^ carbon paste,^9,15^ and others. Although these reported electrodes have been successfully applied for DIP detection, a simpler, more stable, and lower cost electrochemical sensor can be more attractive for application in routine biological analysis. Thus, pyrolytic graphite sheet (PGS) electrodes emerge as an alternative due to theirs relevant characteristics, such as high conductivity, flexibility, and lightweight.^16^ Additionally, an improvement in the electrochemical sensing features of PGS is presented in the literature by applying cold plasma treatment^16−18^ since the treatment promotes the insertion of oxygen groups and edge plane defects into carbonaceous structures, thus providing more available active sites. Therefore, we present the use of the cold oxygen-plasma-treated PGS electrode (GS-O_2_) for the detection and quantification of DIP in synthetic urine samples.

## 2 Experimental Section

### 2.1 Reagents, Samples, and Materials

PGS with 0.07 mm width, 2.5 Ω electrical resistance, and 55.6 S cm^–1^ electrical conductivity was acquired from Panasonic (Mansfield, Texas, USA). Each GS sheet costs $ 17.38 and can generate 180 electrodes; therefore, the cost of each electrode is around $ 0.09.^18^ All solutions were prepared using ultrapure water (*R* ≥ 18 MΩ cm) obtained from a purification system Millipore Direct-Q3 (Bedford, USA). Caffeine (CAF) (99.9%), paracetamol (99.0%), and salicylic acid (99.0%) were obtained from Synth (Diadema, Brazil); acetic (99.7%), ascorbic (99.0%), and phosphoric acids from Vetec (Rio de Janeiro, Brazil); sodium hydroxide (97.0%) from Dinâmica (Diadema, Brazil); boric acid (99.0%) from Appli-chem Panreac (Barcelona, Spain); sodium nitrate from Caal (Araçatuba, Brazil); and citric acid from Sandoz (Cambé, Brazil) from Cinética (Itapevi, Brazil). Calcium chloride dihydrate (99%), sodium chloride (99%), sodium sulfate (99%), potassium phosphate monobasic (99%), potassium chloride (99%), ammonium chloride (99.5%), and urea (URE) (99%) were purchased from Sigma-Aldrich. Argon (99.99%) and O_2_ (99.9%) were purchased from White Martins Co.

All electrochemical measurements were performed in an Autolab PGSTAT302N potentiostat. An oxygen-treated graphite sheet (GS-O_2_) was employed as the working electrode, whereas Ag/AgCl (KCl_SAT_) was used as the reference electrode and a Pt wire as the counter electrode. All electrochemical measurements were performed using a 3D-printed electrochemical cell with a total volume of 10 mL. The cell was constructed by a desktop FDM 3D printer and an ABS filament. More information about the 3D-printed cell can be found in a previously published work.^19^ Both counter and reference electrodes were placed in the 3D-printed cell through the cover, while the working electrode was placed at the bottom of the cell as demonstrated in another work.^20^ In these experiments, 0.12 mol L^–1^ Britton–Robinson (BR) buffer solutions (which is a mixture of boric, acetic, and phosphoric acids, all at 0.04 mol L^–1^) with pH ranging from 2 to 12 were used as the supporting electrolyte. A stock solution (10 mmol L^–1^) of DIP was prepared separately after dissolution in the supporting electrolyte and stored in a refrigerator (5 °C). The synthetic urine sample was prepared following the procedure proposed by Antonin and co-workers.^21^ Synthetic urine consists of 6.8 mmol L^–1^ CaCl_2_·2H_2_O, 51.3 mmol L^–1^ NaCl, 14.2 mmol L^–1^ Na_2_SO_4_, 7.3 mmol L^–1^ KH_2_PO_4_, 26.8 mmol L^–1^ KCl, 18.7 mmol L^–1^ NH_4_Cl, and 0.42 mol L^–1^ URE. An amount of 10 mL of synthetic urine was spiked with 1 mmol L^–1^ DIP. 50 μL of the sample was diluted in the electrochemical cell in 4.95 mL of the same supporting electrolyte. The measurements using the SWV technique were performed, and the presence of DIP was checked by oxidation processes, as observed in previous results.

### 2.2 Electrode Surface Treatment

PGS electrodes were cut into square pieces of 10 cm^2^ and submitted to reactive cold plasma discharges using mixtures of O_2_ and argon controlled by needle valves at 100 and 350 mTorr, respectively, for 2 min each. The reactive plasma here used is composed by a microwave-assisted plasma-enhanced chemical vapor deposition (PECVD) system described elsewhere.^22^ The treated electrodes were assembled as demonstrated in a previous work,^20^ in which the graphite electrode is placed into the electrochemical cell and its area (0.22 cm^2^) is delimited by a rubber O-ring.

## 3 Results and Discussion

### 3.1 Electrochemical Behavior of Antibiotics on GS Electrodes

The effect of plasma treatment of GS electrodes has been previously demonstrated by Pereira et al.^16−18^ Surface characterization has shown that graphene edge layers present in GS electrodes are exposed after cold plasma treatment, which decreases the electrode resistance to the charge transfer. Additionally, a higher surface area and more oxygenated surface functional groups are observed after treatment, and based on these results, the plasma-treated GS electrodes were evaluated for DIP detection.

**Figure 1:**
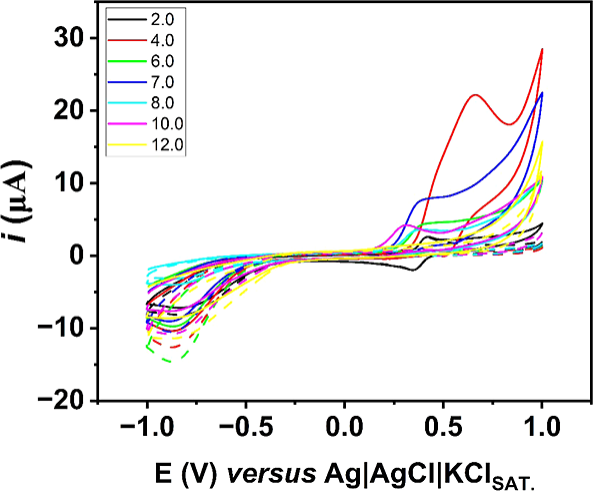
Voltammetric profiles of 200 μmol L^–1^ DIP varying the pH from 2.0 to 12.0 using a 0.12 mol L^–1^ BR buffer solution as the supporting electrolyte at the GS-O_2_ electrode. Dashed lines represent their respective blanks. Scan rate: 50 mV s^–1^; step: 5 mV.

First, the influence of the pH on the electrochemical response of 200 μmol L^–1^ DIP was evaluated using 0.12 mol L^–1^ BR buffer under pH-adjusted values between 2.0 and 12.0 (Figure 1). The chosen medium for the next experiments was pH 4.0, which provided higher peak current and, however, with a slight shift of potentials to regions further from 0.0 V (when compared with higher pH values). This result is in accordance with the literature, in which it is demonstrated that the oxidation peaks are present from pH 5.0 to 8.0.^23^

**Figure 2:**
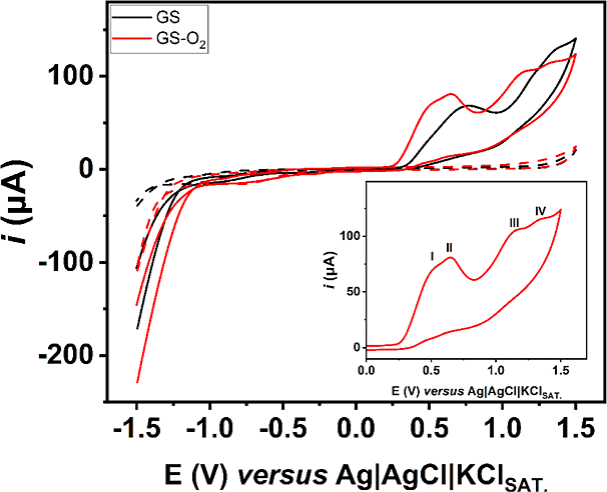
Voltammetric profiles of 1 mmol L^–1^ DIP at GS (black line) and GS-O_2_ (red line) as working electrodes using a 0.12 mol L^–1^ BR buffer solution pH 4 as the supporting electrolyte. The dashed lines represent the respective blanks. Scan rate: 50 mV s^–1^; step: 5 mV. Inset: zoomed cyclic voltammetry (CV) of 1 mmol L^–1^ DIP at GS-O_2_ evidencing the four oxidation peaks.

Also, a cyclic voltammogram was performed using the chosen media (pH = 4.0) in a wider potential window using graphite sheet electrodes both treated (GS-O_2_) and untreated (GS), and the result is presented in Figure 2. It is possible to notice a slight increase in current for all oxidation processes, which is possible due to a higher surface area present in the plasma-treated surface.^17,18^ Additionally, a slight anticipation of the oxidation processes is observed, which can be related to a higher catalytic effect enabled by the graphene edge planes exposed after cold plasma treatment.^17^ A higher exposure of edge planes could also explain the fact that, for the GS electrode, only two processes are visible in the cyclic voltammogram, while four processes can be identified using the GS-O_2_ surface; thus, this electrode was selected for further experiments.

From a mechanistic point of view, it is possible to notice what appears to be a double peak at +0.49 V and at 0.64 V (which in this work will be considered as peak I and peak II, respectively) and peaks at +1.13 V (peak III) and +1.32 V (peak IV). This behavior is also known in the literature, and those peaks are related to the loss of the sulfite group (peak I), which is the easiest step, followed by the oxidation of the methyl group at the outer nitrogen to the methyl amino group (peak II) and finally an oxidation to a methyl-methanal amino or to simple amine.^23^

**Figure 3:**
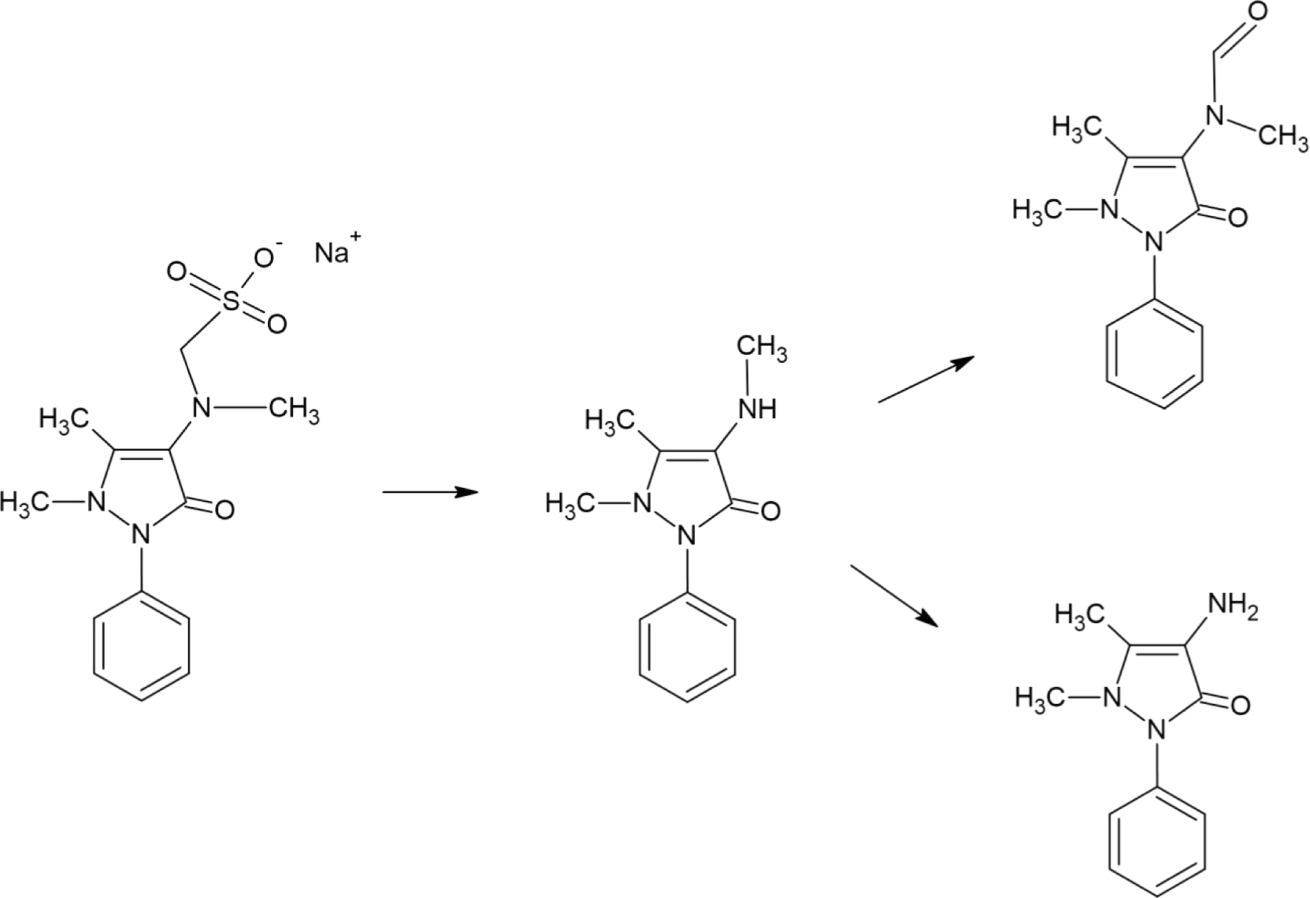
Possible oxidation pathway for DIP electrochemical oxidation.

Bacil and co-workers proposed a reaction pathway after the first oxidation process: the first oxidation process (+0.6 V) is possibly a reversible peak depending on the existence of further processes, and the product is described by them as an iminium cation radical. This intermediate can suffer a nucleophilic attack by water to produce another reactive intermediate; the chemical step after the electrochemical process could be a dimerization or a further electrochemical oxidation. Additionally, they mention that peak II and peak III could be scan rate dependent.^24^ An adaptation of the possible pathway for DIP oxidation is presented in Figure 3.

**Figure 4:**
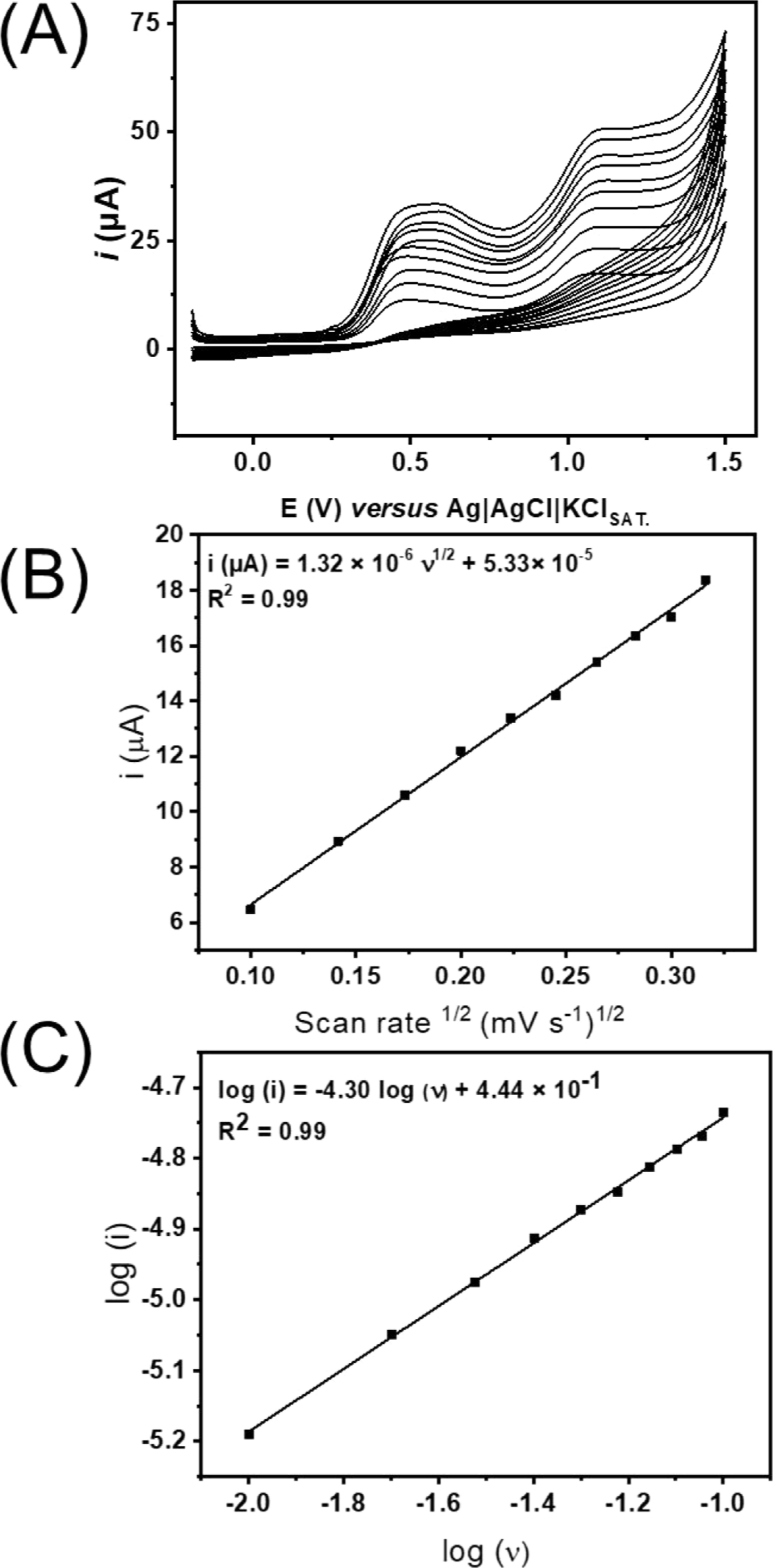
Cyclic voltammograms: O_2_ plasma-treated GS (GS-O_2_) (A) in the presence of 1 mmol L^–1^ DIP using a 0.12 mol L^–1^ BR buffer solution pH 4 as the supporting electrolyte at the scan rates 10, 20, 30, 40, 50, 60, 70, 80, 90, and 100 mV s^–1^, squared root of the scan rate versus peak current (B), and log(*v*) versus log(*i*) (C).

Also, aiming to investigate the mass transport regime involved in the oxidation of DIP toward the proposed surface, CV analyses were carried out at different scan rates (from 10 to 100 mV s^–1^), and the results are presented in Figure 4A–C. Peak I, which is located at around 0.5 V, was selected since it is the first oxidation process, and the other peaks depend on the first one. Then, a linear relationship between the squared root of the scan rate and the peak current was found, indicating that the diffusion of the molecules toward the electrode surface is what controls the reaction rate. Likewise, the log(*i*) versus log(*v*) plot showed a linear adjustment with a slope of 0.44, which confirms the previous statement.^25−28^

### 3.2 Dipyrone Detection Using the GS-O_2_ Electrode by Square Wave Voltammetry

**Figure 5:**
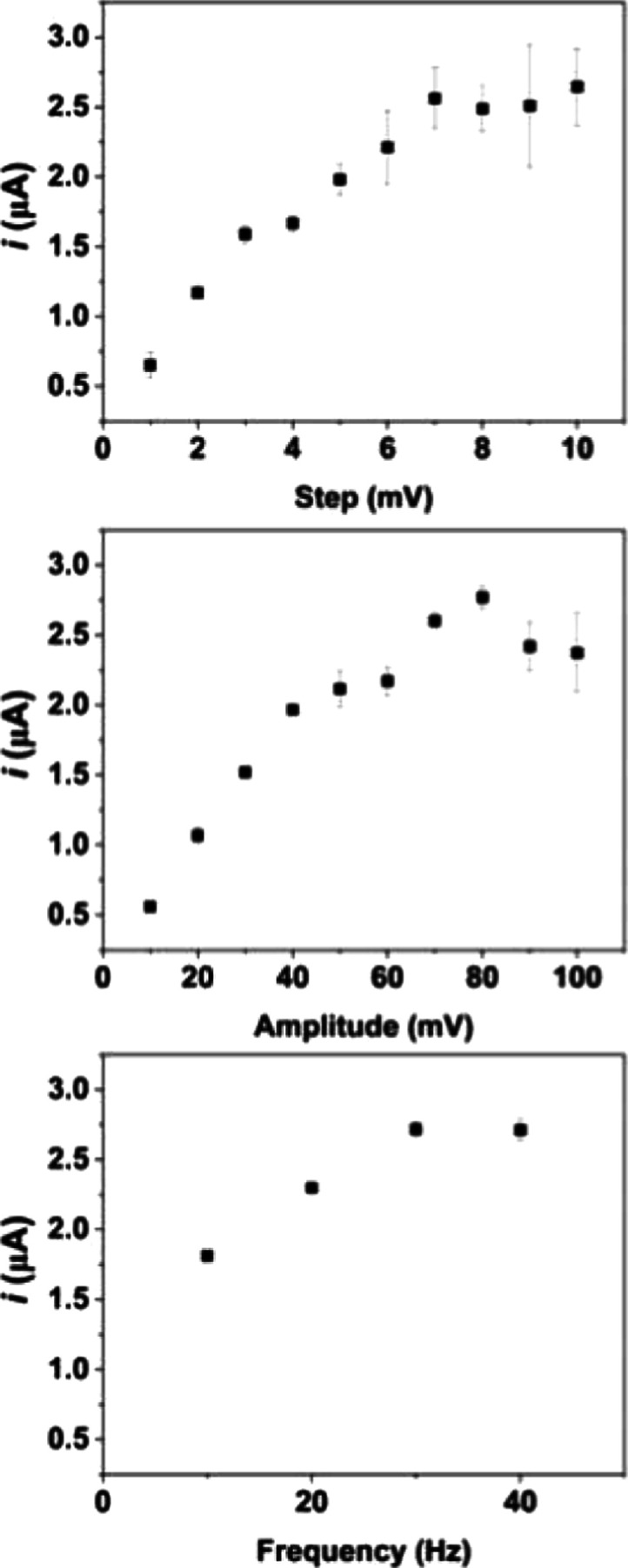
Optimization of SWV parameters for 50 μmol L^–1^ DIP using a 0.12 mol L^–1^ BR buffer solution pH 4, considering peak I. Amplitude, frequency, and step potential (*n* = 3).

Square wave voltammetry (SWV) technique was chosen over CV due to its ability to minimize the contribution of capacitive current,^29^ allowing the detection of species even at low concentrations, which is mandatory for emerging contaminants in routine environmental analyses. SWV parameters, such as step potential, frequency, and amplitude, were properly studied for the DIP first peak at GS-O_2_, aiming for a better analytical response and resolution. The ranges evaluated are as follows: step potential: 1–10 mV; frequency 10–40 Hz (in frequencies higher than 40 Hz, the GS electrode is deteriorated); and amplitude: 10–100 mV. The results are presented in Figure 5. Within this frame, the selected parameters were 7 mV for step potential, 30 Hz for frequency, and 80 mV for amplitude. These values were selected based on analytical response and lower deviation.

Another important aspect when a method was developed to determine DIP is the molecule’s adsorption. Due to the formation of several subproducts, a high adsorption and consequent electrode fouling is observed, which is also in accordance with what is demonstrated in the literature using other carbon electrodes;^23^ however, it is more prominent in higher pH conditions than the selected here. Thus, considering it was intended to correctly measure the presence of DIP in a “clean” surface, a cleaning potential (−1.2 V) was applied for 15 s under stirring conditions before every SWV scan, which renewed the electrode surface between measurements.

**Figure 6:**
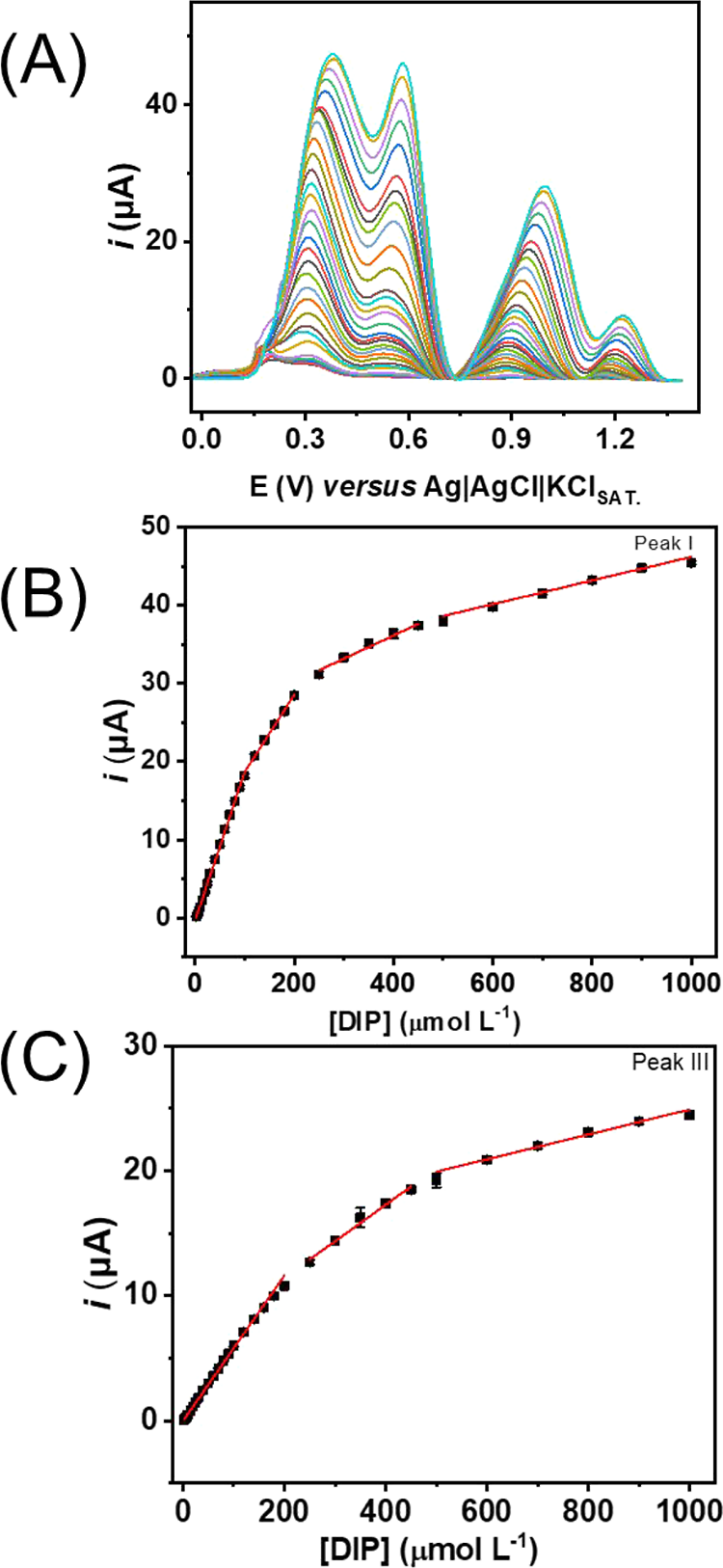
(A) Baseline-corrected SWV responses for increasing concentrations of DIP on GS-O_2_ using a 0.12 mol L^–1^ BR buffer solution pH 4. SWV conditions: 7 mV for step potential, 30 Hz for frequency, and 80 mV for amplitude. (B,C) Linear adjustments for peak I and peak III, respectively.

The calibration curves obtained are presented in Figure 6A–C, with different linear ranges for two different oxidation peaks (peak I and III; peak II did not present a linear behavior). The fact that it is possible to observe linear ranges for both peaks is relevant to a proper qualitative and quantitative detection of DIP in a biological medium and in the presence of possible interferents. Table 1 also summarizes the calibration curve details, such as the linear range and limit of detection (LOD). The LOD values were calculated following the IUPAC recommendation,^30^ where LOD = 3*s*_B_/*S* (*s*_B_ is the standard deviation of ten measurements of the blank and *S* is the slope of the calibration curve). It is possible to notice lower LOD values for both oxidation peaks, which were estimated to be 0.36 μmol L^–1^ for peak I and 0.31 μmol L^–1^ for peak III. Although a higher slope was observed for peak I, a more stable (least variable) measure of the blank was achieved at around +0.9 V (third peak) if compared with the first peak (at +0.3 V), which is possibly responsible for a lower LOD observed for peak III.

**Table 1:** Analytical Parameters Obtained for the Detection of DIP by SWV Using a GS-O_2_ Electrode

analytical parameters	linear range #	peak I	peak III
linear range (μmol L^–1^)	1st, 2nd, 3rd	2.5–100.0, 100.0–200.0, 250.0–450.0	2.5–200.0, 250.0–450.0, 500.0–1000.0
slope (μA L μmol^–1^)	1st, 2nd, 3rd	1.90 × 10^–7^, 9.87 × 10^–8^, 2.97 × 10^–8^	5.82 × 10^–8^, 2.91 × 10^–8^, 9.97 × 10^–9^
*R*^2^	1st, 2nd, 3rd	0.99, 0.99, 0.97	0.99, 0.99, 0.97
LOD (μmol L^–1^)		0.36^a^	0.31^a^

a Calculated based on the first linear range.

Additionally, the adsorbing effect of the oxidation products onto the electrode surface causes the existence of several linear ranges. In this particular case, since the treated PGS electrode possesses a highly porous surface, as mentioned in previous works,^16−18^ more than one linear range can be achieved. Nonetheless, for both observed peaks, a considerably wide first linear range was obtained (2.5–100.0 μmol L^–1^ for peak I and 2.5–200.0 μmol L^–1^ for peak III). Also, the existence of additional linear ranges through the range of 2.5–1000.0 μmol L^–1^ enables the use of this method in several samples, in most cases, even without dilution steps.

The analytical performances of the proposed electrodes were compared with previous reports in terms of linear range and LOD for DIP detection, and Table 2 summarizes the data obtained. It is possible to notice that a few works present a lower LOD if compared with the methodology developed here; however, it is important to emphasize that SPE-BDD is an expensive commercial material, while CD-HPC/CBPE, G-PS, and PtNPs/graphene/GCE present laborious synthesis, which is both time-consuming and relatively expensive. In comparison, the treatment applied in this work is faster (2 min) and cheaper (approximately $0.09 per electrode) and presents a significant decrease in waste from vacuum plasma; thus, it can be an attractive alternative to produce sensitive electrodes in large scale.^16,18,31^ Additionally, a minimal infrastructure is needed to perform the proposed surface treatment since the PECVD system used here was homemade.^22,32^

**Table 2:** Comparison between the Electrodes Proposed in This Work and Previous Reports^a^

electrode	method	LOD (μmol L^–1^)	linear range (μmol L^–1^)	refs
StPE	SWV	0.82	2.0–10.0	(33)
NPAu-μE	SWV	1.20	1.0–200.0	(34)
SPE-BDD	SWV	0.30	2.0–250.0	(2)
CD-HPC/CBPE	SWV	0.009	0.45–22.70	(15)
PtNPs/graphene/GCE	SWV	0.05	1.0–100.0, 100.0–220.0	(35)
G-PS	CV	0.20	0.67–90.0	(36)
NSTFE	chronoamperometry	1.20	1.1–470.0	(37)
Au/GCE	DPV	0.15	2.74–116	(38)
GS-O2	**SWV**	**0.31**	**2.5–200.0**	**this work**

a StPE: stencil-printed electrode using conductive graphite-based; NPAu-μE: nanoporous gold microelectrode arrays; SPE-BDD: screen-printed boron-doped diamond electrode; CD-HPC/CBPE: carbon black paste electrode modified with α-cyclodextrin and hierarchical porous carbon; PtNPs/graphene/GCE: glassy carbon electrode modified with graphene and platinum nanoparticles; G-PS: graphite–polystyrene composite; NSTFE: nickel–salen thin-film-modified electrode; Au/GCE: gold particle-modified glassy carbon electrode.

**Figure 7:**
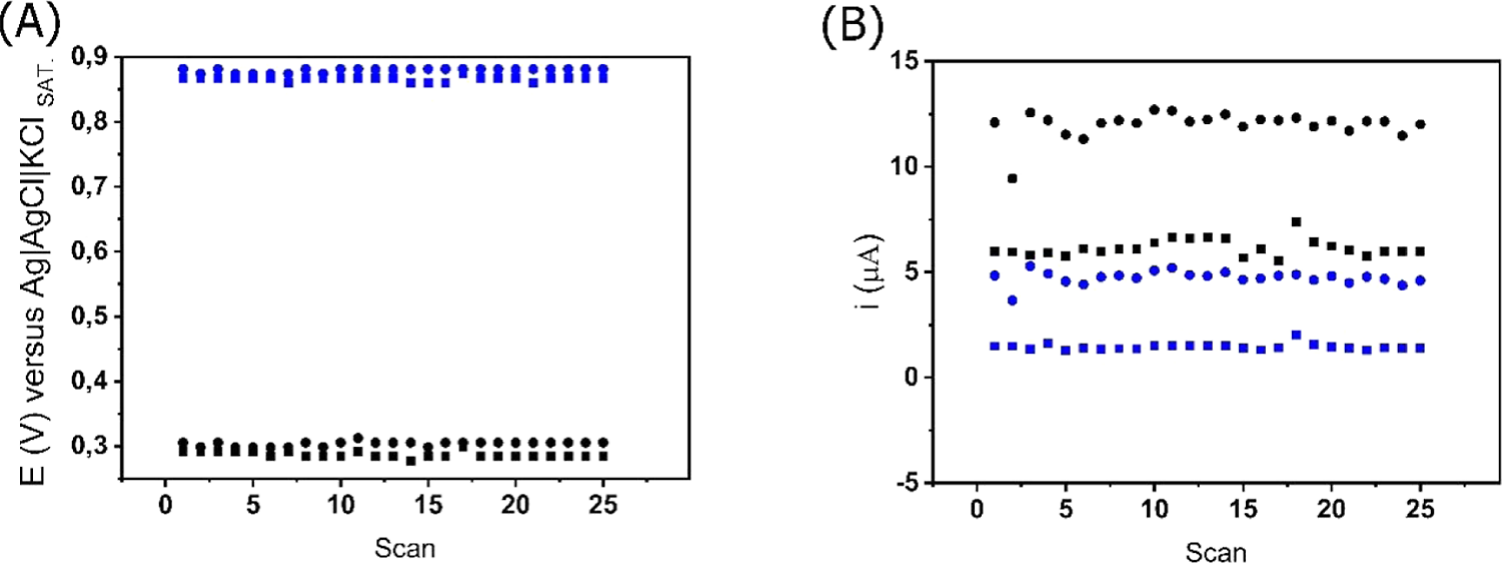
Potential (A) and current (B) responses for consecutive SWV scans on the GS-O_2_ electrode using a 0.12 mol L^–1^ BR buffer solution pH 4 for peak I (black line) and peak III (blue line) in different DIP concentrations (● 100 μmol L^–1^ and ■ 50 μmol L^–1^). SWV conditions: 7 mV for step potential, 30 Hz for frequency, and 80 mV for amplitude.

Additionally, using the SWV technique, consecutive voltammograms (*n* = 25) were recorded to evaluate the electrode stabilities for the detection of DIP. The results (Figure 7A,B) demonstrate that *E*_PEAK_ (for both peak I and peak III) presented RSD below 1.6% for DIP using the GS-O_2_ electrode, both at 50 and 100 μmol L^–1^. Furthermore, considering the oxidation current, the RSD values observed when using the treated surface was lower than 10%, which is described in the literature^39^ as an acceptable deviation value (for both peak I and peak III and for both 50 and 100 μmol L^–1^), confirming that the cold plasma treatment also provides higher stability to the sensing system.

### 3.3 Interferents Study

Unabsorbed DIP is commonly found in urine; thus, several other urine contents can interfere in the electrochemical response, and therefore, the behavior of DIP in the presence of such contaminants was also evaluated. The possible interferents studied were glucose (GLU), ascorbic acid (AA), urea (URE), cations (Mg^2+^, Na^+^, Ca^2+^, and NH_4_^+^), and anions (Cl^–^, SO_4_^2–^, CO_3_^2–^, HCO_3_^–^, and NO_3_^–^) according to previous works.^40^ The results are presented in Figure 7. DIP was added at 50 μmol L^–1^, while the studied interferents were added at 500 μmol L^–1^ (10 times higher). The interference was evaluated as for the oxidation potential shift (vs Ag|AgCl|KCl reference electrode) and current variation as a percentage [(analyte + interferent current/analyte current) × 100],^17^ and the results are presented in Table 3.

**Table 3:** Effect of the Possible Interferents on Dipyrone Current and Potential^a^

interferent	peak I (0.34 V versus Ag|AgCl|KCl_SAT_)		peak III (0.96 V versus Ag|AgCl|KCl_SAT_)	
	current (%)	potential (%)	current (%)	potential (%)
GLU	+18.3	+2.2	+1.7	0.0
AA	+1103.6	+7.7	–19.5	+1.0
URE	+18.3	0.0	–1.7	0.0
uric acid (UA)	–19.6	–0.5	–10.5	0.0
CAF	–30.6	+3.4	–4.2	+0.8
Mg^2+^	+30.2	+1.6	+1.7	0.0
Na^+^	–5.7	+1.1	–2.4	+0.8
Ca^2+^	+48.4	–2.7	–6.1	–0.4
NH_4_^+^	+22.2	–1.1	–0.04	–0.4
Cl^–^	–5.7	+1.1	–2.4	+0.8
SO_4_^2–^	+30.2	+1.6	+1.7	0.0
CO_3_^2–^	+48.4	–2.7	–6.1	–0.4
HCO_3_^–^	+22.2	–1.1	–0.04	–0.4
NO_3_^–^	+14.2	+1.1	+133.8	+0.8

a The % values correspond to [(analyte current + interferent current/analyte current) × 100].

In fact, if considering the first oxidation peak, almost every studied interferent substantially changes the current response registered for DIP, either to higher or lower values; thus, the quantification considering peak I is only reliable in the presence of Na^+^ and Cl^–^. However, for all molecules/ions studied, the potential deviation was below 10%, which means that it is possible to identify the presence of DIP. On the other hand, the most important aspect of the results found is what was observed for peak III. GLU, URE, CAF, Mg^2+^, Na^+^, Ca^2+^, NH_4_^2+^, Cl^–^, SO_4_^2–^, CO_3_^2–^, and HCO_3_^–^ presented current and potential deviations below 10%, which, again, means that in the presence of those species, it is possible to both identify and quantify the presence of DIP.

For UA, the current variation was slightly above 10%, which means that a slight error can be obtained for quantification of DIP in the presence of UA; however, its identification can be successfully proceeded. It is important to notice that this experiment was performed with the interferents present in a concentration 10 times higher than DIP concentration; yet, previous studies point that, for a healthy adult, UA is present in urine in concentrations of 4.03 ± 0.95 mg L^–1^,^41^ which is significantly lower than DIP concentration in urine.^42^ AA also displayed a considerable fluctuation in current for peak III (19.5%), and this effect is widely discussed in the literature^43,44^ since AA compromises urinary results for several other analytes. Nonetheless, although DIP quantification may present errors, its identification is still reliable.

Additionally, NO_3_^–^ displayed a high deviation in current for peak III, which could be associated with the interaction of anions with the active sites of the carbonaceous surface, promoting an adsorption phenomenon. Then, a competitive behavior between DIP and nitrate occurs on the PGS surface, favoring an easy adsorption of nitrate due to its dimensions and binding groups and consequently altering the linear relationship of the DIP determination.

### 3.4 Dipyrone Determination in Synthetic Urine Samples

Using the previously selected SWV conditions, GS-O_2_ was used as a working electrode for DIP determinations in synthetic urine samples. Although DIP rapidly hydrolyzes and consequently passes through several reactions after application in human body (the possible molecules formed are 4-methylaminoantipyrine, 4-aminoantipyrine, 4-formylaminoantipyrine, 4-acetylaminoantipyrine, arachidonoyl-4-methylaminoantipyrine, and arachidonoyl-4-aminoantipyrine),^5^ the total absorption varies from 54% to 89% depending on the method of administration (tablets, drops, suppositories, intramuscular, and intravenous injection). This means that, in a 1 g administration, up to 460 mg (1.48 mmol) of DIP could be excreted from the human body.^42^

**Figure 8:**
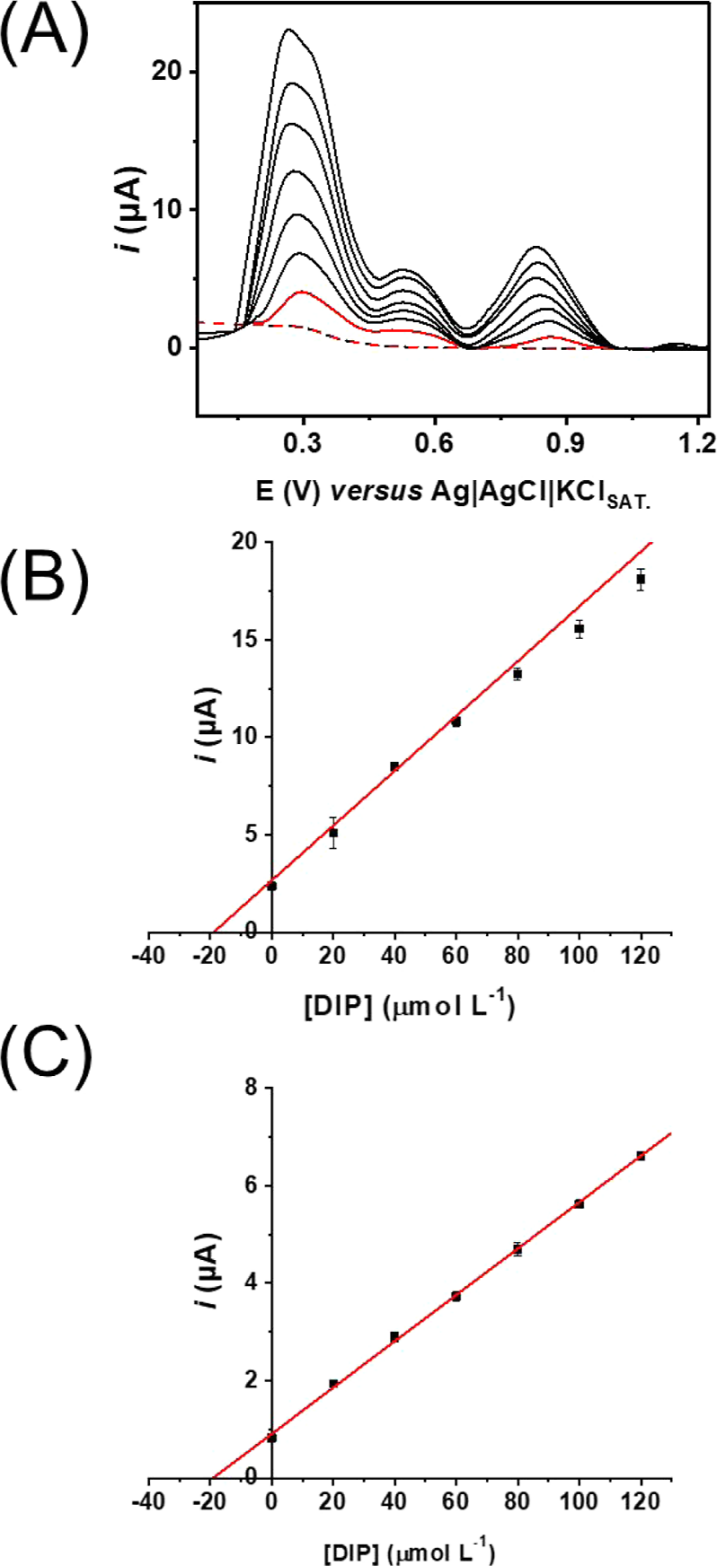
(A) Baseline-corrected SWV of synthetic urine samples (red dotted line), spiked with DIP (red line) using GS-O_2_ as the working electrode, and standard additions of 20 μmol L^–1^ (black lines). The synthetic urine was diluted 5 times. SWV conditions: 7 mV for step potential, 30 Hz for frequency, and 80 mV for amplitude. (B) Standard addition curve for peak I and (C) peak III.

Thus, the synthetic urine samples were spiked with 100 μmol L^–1^ DIP and then diluted 5 times into a 10 mL electrochemical cell containing 0.12 mol L^–1^ BR buffer solution pH 4. Then, six standard additions of 20 μmol L^–1^ DIP were made, and the square wave voltammograms are presented in Figure 8A. The standard addition curves for peak I and peak II are presented in Figure 8B,C, respectively, and Table 4 summarizes the results of recovery of DIP in spiked synthetic urine samples. In this case, three different experiments in the same assembly were performed, and the recovery was evaluated for both peak I and peak III. For peak I, a recovery of 96.4 ± 8.3% was observed, and for peak III, 87.5 ± 13.7%, which demonstrate that the matrix effect does not affect the DIP determination, and this electrode can be applied as a simple and portable way to analyze real samples.

**Table 4:** Results Obtained from the Recovery Studies in Synthetic Urine Samples Spiked with DIP (*n* = 3)

	spiked (μmol L^–1^)	found (μmol L^–1^)	%
peak I	20.0	19.3 ± 1.7	96.4 ± 8.3
peak III	20.0	17.5 ± 2.7	87.5 ± 13.7

## 4 Conclusions

We demonstrated that the oxygen plasma-treated electrode can be successfully used to identify the presence and quantify the amount of DIP in synthetic urine, as well as in the presence of possible interferents. This is attributed to the increased surface area and especially to the structural disorder and presence of oxygen functional groups, which confer electrocatalytic effects. Additionally, a low LOD was found, compared with other electrodes found in the literature, which demonstrates the high applicability of this treatment method (cold plasma treatment is a reagentless process that takes only 2 min). The combination of PGS with cold plasma treatment allows the mass production of low-cost, highly sensitive and reproductive, malleable, and disposable electrodes, which can be used on a large scale for different analysis.
